# Dietary Patterns, Cooking Methods, and Their Association with Prediabetes Risk Markers in Romanian University Students: A Cross-Sectional Analysis

**DOI:** 10.3390/nu18060977

**Published:** 2026-03-19

**Authors:** Teodora Piroș, Raluca Lupusoru, Lavinia Cristina Moleriu, Călin Muntean, Radu Dumitru Moleriu, Dora Mihalea Cîmpian, Mădălina Gabriela Cincu, Elena Gabriela Strete, Amalia Gabriela Timofte, Ruxandra-Cristina Marin

**Affiliations:** 1 Department III, Functional Science, Discipline of Medical Informatics and Biostatistics, “Victor Babes” University of Medicine and Pharmacy, 300041 Timisoara, Romania; teodora.piros@student.umft.ro (T.P.); raluca.lupusoru@umft.ro (R.L.); radu.moleriu@umft.ro (R.D.M.); amalia.timofte@student.umft.ro (A.G.T.); 2 Center for Modeling Biological Systems and Data Analysis, “Victor Babes” University of Medicine and Pharmacy, 300041 Timisoara, Romania; 3 Gastroenterology and Hepatology Clinic, County Emergency Hospital “Pius Brinzeu”, 300723 Timisoara, Romania; 4 Department of Ethics and Social Sciences, George Emil Palade University of Medicine, Pharmacy, Science and Technology of Târgu Mureş, 540139 Târgu Mureş, Romania; dora.cimpian@umfst.ro; 5 Doctoral School of Faculty of Medicine, George Emil Palade University of Medicine, Pharmacy, Science and Technology of Târgu Mureş, 540142 Târgu Mureş, Romania; cincu.madalina@yahoo.com; 6 Department of Psychiatry, George Emil Palade University of Medicine, Pharmacy, Science and Technology of Târgu Mureş, 540139 Târgu Mureș, Romania; elena.buicu@umfst.ro; 7 Discipline of Pharmacology, Clinical Pharmacology and Pharmacotherapy, “Carol Davila” University of Medicine and Pharmacy, 050474 Bucharest, Romania; ruxandra.marin@umfcd.ro; 8 Doctoral School of Biological and Biomedical Sciences, University of Oradea, 410087 Oradea, Romania

**Keywords:** prediabetes, HbA1c, students, dietary patterns, fast-food consumption, ultra-processed foods, cooking methods, body mass index, triglycerides/HDL ratio

## Abstract

**Background**: Young adulthood represents a critical period for the emergence of early metabolic disturbances, potentially influenced by dietary shifts toward convenience and ultra-processed foods. However, evidence linking dietary patterns and cooking practices with objective metabolic biomarkers in Romanian university students remains limited. **Methods**: This cross-sectional study included 693 students aged 18–24 years at the Victor Babeș University of Medicine and Pharmacy, Romania (June–July 2025). Dietary habits, food preferences, and cooking practices were assessed using a structured online questionnaire, while anthropometric and biochemical data were obtained from university health records. The primary outcome was glycated hemoglobin (HbA1c), a marker of average blood glucose levels over the previous 2–3 months. Prediabetes was defined as HbA1c 5.7–6.4%. Dietary patterns were identified using k-means clustering based on fast-food consumption frequency, main meal of the day, fruit and vegetable intake frequency, and predominant cooking method. Multivariable regression models assessed associations between dietary variables and glycemic or lipid outcomes. **Results**: Prediabetes prevalence was 21.1% (diabetes: 1.4%). Three dietary patterns were identified: health-conscious (prediabetes 15.4%), mixed (20.0%), and fast-food oriented (27.3%; χ^2^ *p* = 0.003). Fast-food consumption frequency was independently associated with higher prediabetes risk (OR = 1.78 per category; 95% CI 1.38–2.30; *p* < 0.001) and higher HbA1c levels (β = 0.147; *p* < 0.001), while fruit and vegetable intake showed an inverse association with HbA1c (β = −0.109; *p* < 0.001). A dose–response relationship was observed between fast-food frequency and both HbA1c and prediabetes prevalence (*p*-trend < 0.001). An interaction between high-temperature cooking methods and frequent fast-food consumption was observed for HbA1c (*p* = 0.023). BMI and sex were the strongest predictors of lipid outcomes, although fast-food intake was associated with higher triglyceride levels (*p* = 0.034). **Conclusions**: Among Romanian university students, dietary patterns characterized by frequent fast-food consumption were associated with higher HbA1c levels and greater prediabetes prevalence. A high-temperature cooking method was associated with higher glycemic levels when combined with frequent fast-food intake. These findings suggest that early dietary behaviors during university years may be relevant for metabolic risk profiles in young adults.

## 1. Introduction

Type 2 diabetes mellitus (T2DM) is a major global health burden with rapidly rising prevalence worldwide. Using International Diabetes Federation (IDF) Atlas-based modeling, global diabetes prevalence in adults aged 20–79 years was estimated at 536.6 million in 2021 and is projected to reach 783.2 million by 2045, reflecting the combined impact of demographic change, urbanization, and lifestyle-related risk factors [1].

A particularly concerning trend is the growing recognition of early-onset T2DM (often defined as onset before 40 years), which is associated with more aggressive disease trajectories and earlier complication burden compared with later-onset T2DM. Recent reviews highlight that incidence and prevalence of T2DM in younger individuals have increased substantially in recent decades, with multifactorial drivers including obesity severity, family history, and adverse early-life exposures [2,3,4].

Prediabetes (intermediate hyperglycemia below the diagnostic threshold of diabetes) represents a key prevention window because progression risk is elevated, yet behavioral interventions can meaningfully reduce progression to T2DM. Contemporary clinical guidance emphasizes structured lifestyle approaches, including dietary improvement, physical activity, and weight management, for diabetes prevention in individuals with prediabetes [5].

Supporting this, systematic reviews and meta-analyses in prediabetes populations show that structured lifestyle interventions reduce incident T2DM and improve glycemic trajectories [6,7]. Evidence from landmark prevention trials, such as the Diabetes Prevention Program, demonstrated that intensive lifestyle intervention reduced diabetes incidence by approximately 58% among high-risk individuals [8]. More recent meta-analytic evidence confirms that lifestyle interventions significantly reduce the risk of developing T2DM and improve glycated hemoglobin (HbA1c) and glucose regulation in individuals with prediabetes [6,9]. These findings reinforce the importance of early behavioral interventions during the prediabetes stage as a key strategy for diabetes prevention.

Young adulthood represents a particularly important period for implementing such preventive strategies, as lifestyle habits established during this life stage may influence long-term metabolic trajectories. The transition into university life commonly coincides with reduced routine, time constraints, financial limitations, and limited cooking resources, conditions that can promote irregular meal patterns, greater reliance on convenience foods, and lower intake of fruits and vegetables. Evidence in student populations continues to document frequent breakfast skipping, irregular eating patterns, low fruit and vegetable intake, and sedentary behaviors relevant to early cardiometabolic risk [10].

This is clinically important because adverse glycaemic profiles can occur even without overt obesity, and cardiometabolic risk may not be fully captured by BMI alone. Evidence shows that individuals with normal or moderately elevated BMI may still exhibit metabolic disturbances (insulin resistance, dyslipidaemia, or impaired glucose regulation) often described as the metabolically unhealthy normal-weight phenotype [11,12,13]. These findings highlight the limitations of relying exclusively on BMI as a marker of metabolic risk and support the importance of evaluating metabolic biomarkers when assessing early cardiometabolic vulnerability.

Dietary behaviors represent one of the most important modifiable determinants of metabolic health during this life stage. Dietary pattern approaches provide a more realistic representation of habitual intake than single-nutrient analyses. Western-type dietary patterns, typically higher in refined grains, processed foods, and sugar-sweetened beverages, have consistently been linked to higher T2DM risk [14,15].

In parallel, the expanding literature on industrially processed diets indicates that higher ultra-processed food (UPF) consumption is associated with increased T2DM risk in dose–response meta-analytic evidence [16].

Beyond dietary composition, how food is prepared may also influence metabolic outcomes. High-temperature cooking methods (e.g., deep-frying or griddling) increase exposure to thermal processing byproducts and may cluster with lower diet quality. A prospective analysis reported higher T2DM risk with greater consumption of deep-fried foods and suggested that substituting fried or griddled preparations with boiled or steamed foods was associated with lower risk [17].

From a biological standpoint, cooking methods represent an additional dimension of dietary quality that may influence metabolic health. High-temperature cooking techniques promote the formation of advanced glycation end products (AGEs) and lipid oxidation products, which can induce oxidative stress, activate inflammatory pathways, and impair insulin sensitivity [18,19,20,21].

Oxidative stress is increasingly recognized as a central mediator linking metabolic disturbances, inflammation, and tissue injury in metabolic disease contexts [22].

In contrast, lower-temperature cooking methods such as boiling, steaming, and baking are associated with reduced AGE formation and better preservation of nutrient integrity [18,19,20,21].

Beyond metabolic outcomes, emerging evidence suggests that diet-related metabolic disturbances may also have implications for mental and cognitive health. Meta-analytic evidence indicates that higher ultra-processed food consumption is associated with greater odds of depressive and anxiety symptoms and higher subsequent depression risk [23,24].

These observations suggest that dietary patterns associated with metabolic dysregulation may also influence broader aspects of health, including psychological well-being and cognitive functioning. This further underscores the importance of examining dietary behaviors early in adulthood when long-term metabolic trajectories begin to develop.

Metabolic dysregulation itself may also influence cognitive function. Studies integrating ambulatory glucose assessment and insulin resistance markers suggest that glucose regulation relates to cognitive domain performance through pathways including inflammation, vascular dysfunction, and altered brain insulin signaling [25].

Emerging evidence indicates that insulin resistance and prediabetes may affect cognitive health even before overt diabetes develops. A meta-analysis of prospective cohort studies examining prediabetes and incident dementia highlights the relevance of early dysglycaemia for Alzheimer’s disease-related outcomes [26], while longitudinal studies suggest that higher HbA1c trajectories may be associated with faster cognitive decline [27].

From a pathophysiological perspective, insulin acts as a neuromodulator in the brain, with receptors distributed in hippocampal and cortical circuits involved in learning and memory. Impaired central insulin signaling (“brain insulin resistance”) has been linked to mitochondrial dysfunction, neuroinflammation, and pathways implicated in cognitive decline and Alzheimer’s disease [28,29]. These mechanisms provide a biological framework linking metabolic disturbances, including insulin resistance and early glycaemic alterations, with potential effects on brain health. Accordingly, identifying modifiable lifestyle factors (dietary patterns and food preparation practices) that contribute to early metabolic dysregulation is an important area of investigation. In parallel, hyperglycaemia-related oxidative stress and accumulation of advanced glycation end products may contribute to neuroinflammation and neurodegenerative vulnerability [30].

Collectively, these converging findings suggest that early metabolic dysregulation may contribute to broader health vulnerabilities. Consequently, identifying modifiable lifestyle factors influencing metabolic health in early adulthood represents an important prevention opportunity. Dietary strategies that improve metabolic health may therefore also support emotional and cognitive resilience; for example, randomized-trial evidence suggests Mediterranean-style dietary interventions can reduce depressive symptom severity in adults [31].

Despite growing international evidence, limited biomarker-based research has examined how dietary behaviors and food preparation practices relate to early metabolic risk in young adult populations in Eastern European settings. Romania, similar to several Eastern European countries, has experienced substantial shifts in food environments and dietary behaviors, with increasing accessibility of convenience and fast-food options in urban areas and around university campuses. The International Diabetes Federation estimated that 8.4% of Romanian adults were living with diabetes in 2021, corresponding to approximately 1.2 million individuals [32], while global development indicators suggest prevalence around 7.5% in 2024 [33].

European dietary surveillance indicates that Romania is part of a broader regional nutrition transition characterized by increasing availability of packaged and ultra-processed foods. In a cross-European analysis of ultra-processed food intake, Romania showed a measurable contribution of such products to total dietary energy intake (e.g., 15.8% among women in the dataset) [34].

University students may be particularly exposed to these pressures due to time constraints, limited cooking facilities, and the density of inexpensive fast-food outlets near campuses, with Romanian student-focused studies describing suboptimal eating behaviors in university settings [35].

However, objective biomarker-linked evidence integrating dietary patterns and cooking practices in Romanian university students remains limited, particularly regarding early glycemic alterations captured by HbA1c, a biomarker reflecting average blood glucose levels over approximately the previous 2–3 months.

Therefore, the aim of this study was to examine the relationships between habitual dietary patterns, cooking methods, and early metabolic risk markers in a cohort of Romanian university students. Using dietary pattern analysis and multivariable modelling, we evaluated whether fast-food consumption frequency and commonly used cooking practices were associated with glycaemic status, as assessed by HbA1c, and with lipid parameters, while accounting for body mass index and other relevant covariates. We further explored whether preferences for food type, including frozen versus fresh foods, were associated with differences in lipid profiles. The analyses were designed to characterize early metabolic alterations in an apparently healthy young adult population rather than to infer causal relationships.

## 2. Materials and Methods

### 2.1. Study Design and Participants

This cross-sectional study was conducted among students enrolled at the Victor Babeș University of Medicine and Pharmacy, Romania. A convenience sample of 693 students (402 females and 291 males), aged 18–24 years, participated in the study between June and July 2025. The age of participants ranged from 18 to 24 years, reflecting the age distribution of students who completed the questionnaire.

Eligibility criteria included current enrollment as a university student, age ≥ 18 years, and the ability to independently complete the questionnaire. Exclusion criteria included a prior diagnosis of diabetes mellitus, incomplete or inconsistent questionnaire responses, as well as underweight students (BMI < 18.5 kg/m^2^), who represented less than 2% of the initial sample, were excluded from the study prior to statistical analysis.

Participation was voluntary, and all respondents were informed about the study objectives, procedures, and intended use of the data prior to participation. The study protocol was approved by the institutional Ethics Committee of the Victor Babeș University of Medicine and Pharmacy Timișoara (approval no. 95/4 October 2021) and was conducted in accordance with the Declaration of Helsinki and the principles of good biomedical research practice.

### 2.2. Data Collection

Data were collected using a structured, self-administered online questionnaire distributed through university email systems and social media platforms. The questionnaire (File S1) began with an introductory information section describing the study objectives, voluntary nature of participation, confidentiality safeguards, and the intended use of the collected data. Informed consent was obtained at the beginning of the questionnaire, with participants indicating their agreement by recording the date and providing written confirmation.

The questionnaire collected information on demographic characteristics, dietary habits, food preferences, and cooking practices. Dietary variables included the frequency of fast-food consumption (never, rarely, 1–2 times/week, ≥3 times/week); the main meal of the day (breakfast, lunch, or dinner); the frequency of fruit and vegetable consumption, assessed by asking participants how often they consumed fruits and vegetables during a typical week (frequency per week; ordinal scale); food preference (fresh vs. frozen); and the predominant cooking method used at home (boiling, baking, grilling, frying, or other). For statistical analyses, dietary frequency variables were coded as ordinal scores. Fast-food consumption frequency was coded as 0 = never, 1 = rarely, 2 = 1–2 times/week, and 3 = ≥3 times/week. Fruit and vegetable intake frequency was similarly coded as an ordinal variable reflecting increasing weekly consumption. Cooking method was categorized as frying versus other methods (boiling, baking, grilling, or other) in regression analyses.

Anthropometric and biochemical measures were based on student self-reported data. Height and weight were used to calculate body mass index (BMI, kg/m^2^). Blood test results included HbA1c, total cholesterol, low-density lipoprotein (LDL) cholesterol, high-density lipoprotein (HDL) cholesterol, and triglycerides. Prediabetes was defined as HbA1c values between 5.7% and 6.4%, following the criteria of the American Diabetes Association. While some variability in weight and biochemical markers may occur over time—particularly due to lifestyle changes associated with university life—the interval between the medical visit and survey administration was relatively short (typically less than 12 months), making these measurements reasonably representative of the participants’ status at the time of the survey.

All data were handled confidentially, and no personally identifiable information was collected. Responses were anonymized and analyzed in aggregated form.

### 2.3. Statistical Analysis

Descriptive statistics were calculated for all variables. Continuous variables are presented as mean ± standard deviation, and categorical variables are presented as frequencies and percentages.

#### 2.3.1. Dietary Pattern Identification

K-means cluster analysis was employed to identify distinct dietary patterns based on fast-food consumption frequency, main meal of the day, fruit and vegetable intake frequency, and predominant cooking method. The optimal number of clusters was determined using the elbow method and silhouette analysis ($\sum_{j=1}^{k}w_{ij}=1$).

#### 2.3.2. Logistic Regression for Prediabetes

Binary logistic regression was performed to examine independent predictors of prediabetes status (HbA1c ≥ 5.7% vs. <5.7%). Candidate predictors included fast-food consumption score, primary cooking method (frying vs. other), fruit and vegetable intake frequency, BMI, sex, age group (21–24 vs. 18–20 years), defined to approximate earlier versus later stages of university study. Age was categorized to facilitate interpretation and to account for potential lifestyle differences between early and later university years within this relatively narrow age range. Odds ratios (ORs) and 95% confidence intervals (CIs) were reported.

$$ln\left(\frac{P(Y=1)}{1-P(Y=1)}\right)=\beta_{0}+\beta_{1}X_{1}+\beta_{2}X_{2}+\beta_{3}X_{3}+\beta_{4}X_{4}+\beta_{5}X_{5}+\epsilon$$

$$P(Y=1)=\frac{1}{1+e^{-(\beta_{0}+\beta_{1}X_{1}+\ldots +\epsilon )}}$$

where *Y* represents prediabetes status (1 = HbA1c ≥ 5.7%, 0 = normal).

#### 2.3.3. Multivariable Linear Regression with Continuous Outcomes

In addition to logistic regression, multivariable linear regression models were fitted using HbA1c (%) as a continuous dependent variable. Independent variables included fast-food consumption frequency (ordinal, 0–3), primary cooking method (frying vs. other), fruit and vegetable intake frequency (ordinal, 0–3), BMI (continuous, kg/m^2^), sex (male vs. female), and age group (21–24 vs. 18–20 years). Standardized regression coefficients (β) were calculated to facilitate comparison of the relative contribution of each predictor.

Separate multivariable linear regression models were also fitted for each lipid parameter (total cholesterol, LDL-C, HDL-C, and triglycerides) as dependent variables, adjusting for fast-food consumption frequency, cooking method, fruit and vegetable intake, BMI, sex, and age group. The triglyceride-to-HDL cholesterol (TG/HDL) ratio was calculated and analyzed as a secondary outcome reflecting insulin resistance.

#### 2.3.4. Dose–Response Analyses

A dose–response analysis was conducted for fast-food consumption frequency. Trend tests were performed by modeling fast-food intake as an ordinal variable (0 = never, 1 = rarely, 2 = 1–2 times/week, 3 = ≥3 times/week) in linear regression models for HbA1c and triglycerides. The Cochran–Armitage trend test was used to assess linear trends in prediabetes prevalence across fast-food consumption categories.

#### 2.3.5. Stratified and Interaction Analyses

Stratified analyses were conducted by sex (female vs. male) and BMI category (normal weight: BMI < 25 kg/m^2^ vs. overweight/obese: BMI ≥ 25 kg/m^2^). HbA1c levels, lipid parameters, the TG/HDL ratio, and prediabetes prevalence were compared across dietary pattern clusters within each stratum using one-way ANOVA and chi-square tests, as appropriate.

To evaluate potential effect modification, interaction terms (dietary cluster × sex; cooking method × BMI category) were included in multivariable regression models. Interaction analyses between cooking method (frying vs. other) and fast-food consumption frequency (high: ≥1–2 times/week vs. low: rarely/never) were conducted using multiplicative interaction terms in multivariable linear regression models.

#### 2.3.6. Mediation Analysis

Mediation analysis was performed to test whether the association between fast-food consumption and HbA1c was mediated by BMI. The indirect effect was calculated as the product of path coefficients (a × b), with 95% confidence intervals derived from 5000 bootstrap iterations. The direct effect (c′) and total effect (c) were also estimated.

#### 2.3.7. Sensitivity Analyses and Additional Covariates

Sensitivity analyses were performed by repeating all primary models after excluding participants with HbA1c ≥ 6.5% to ensure that observed associations reflected early metabolic alterations rather than effects driven by individuals with overt diabetes.

Food preference (fresh vs. frozen) was additionally included as a covariate in multivariable models assessing lipid and glycemic outcomes to evaluate its independent contribution beyond dietary pattern classification.

All analyses were conducted using R (v. 4.3.2; R Foundation for Statistical Computing, Vienna, Austria) and JASP (v. 0.18.3; JASP Team, University of Amsterdam, Amsterdam, The Netherlands). Statistical significance was set at *p* < 0.05 for all tests.

## 3. Results

### 3.1. Participant Characteristics

The study sample comprised 693 university students with a mean age of 20.8 years (SD = 1.4). Females accounted for 58.6% of participants, and 58.0% were aged 21–24 years. Underweight students (BMI < 18.5 kg/m^2^, <2% of the initial sample) were excluded from the study prior to statistical analysis. Among the remaining participants, the mean body mass index (BMI) was 23.96 kg/m^2^ (SD = 3.86), with 27.4% classified as overweight or obese (BMI ≥ 25 kg/m^2^) and 71% as normal weight (BMI 18.5–24.9 kg/m^2^), according to World Health Organization criteria.

The overall prevalence of prediabetes (HbA1c 5.7–6.4%) was 21.1%, while diabetes (HbA1c ≥ 6.5%) was identified in 1.4% of participants. Mean lipid and glycemic values were within generally accepted reference ranges at the population level, although substantial interindividual variability was observed (Table 1).

### 3.2. Dietary Patterns, Cooking Methods, and Glycemic Markers

**Figure 1:**
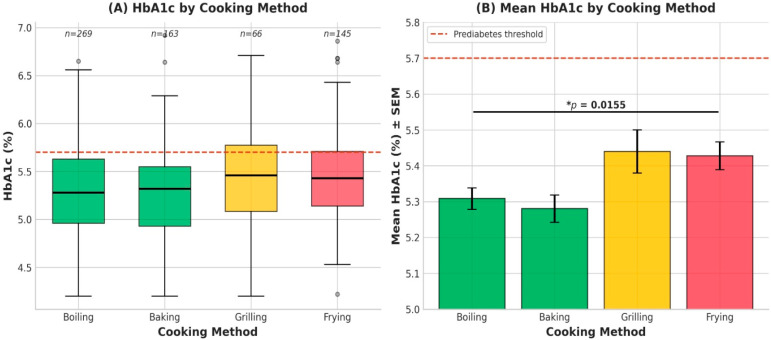
HbA1c levels by primary cooking method. (**A**) Boxplot showing the distribution of HbA1c. (**B**) Bar chart of mean HbA1c with SEM, highlighting the significant difference between high-heat and low-heat methods.

High-temperature cooking methods were associated with higher HbA1c levels. Mean HbA1c was higher among students who primarily used grilling (5.44%) and frying (5.43%) compared with those predominantly using boiling (5.27%) (ANOVA *p* = 0.0155). The distribution of HbA1c values across cooking methods is illustrated in Figure 1A, while corresponding mean values with standard error of the mean are presented in Figure 1B (Figure 1).

**Figure 2:**
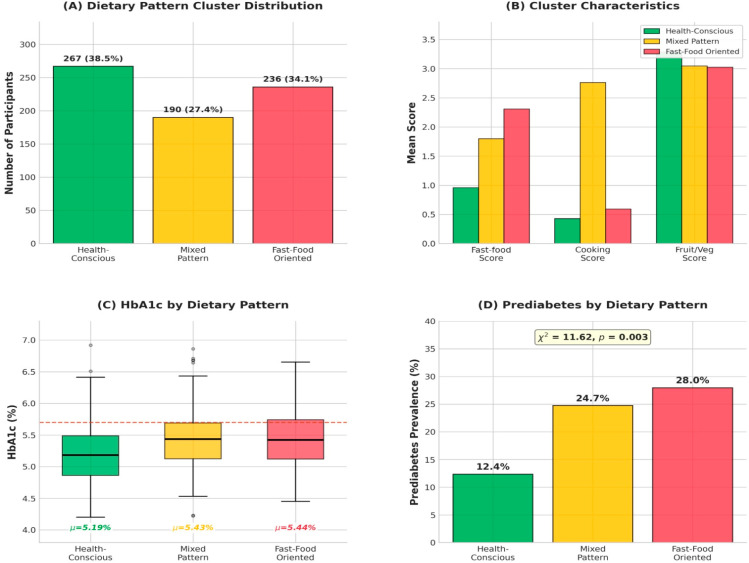
Dietary pattern cluster analysis. (**A**) Distribution of participants across the three clusters. (**B**) Mean scores of key variables defining each cluster. (**C**) Boxplots of HbA1c by cluster. (**D**) Bar chart of prediabetes prevalence by cluster, showing a significant association (χ^2^ = 11.62, *p* = 0.003). Dietary variables were coded as ordinal frequency scores, with higher values indicating higher consumption frequency.

Because cooking practices co-occur with broader dietary behaviors, dietary pattern analysis was subsequently performed to capture habitual combinations of food choices and preparation methods. K-means cluster analysis identified three distinct dietary patterns (Figure 2). The Health-Conscious cluster (n = 272, 39.2%) was characterized by low fast-food consumption, predominant use of boiling, and high fruit and vegetable intake, and exhibited the lowest prediabetes prevalence (15.4%). The Mixed Pattern cluster (n = 150, 21.6%) showed moderate fast-food intake with baking and grilling as common cooking methods and had a prediabetes prevalence of 20.0%. The Fast-Food Oriented cluster (n = 271, 39.1%) was characterized by frequent fast-food consumption, predominant frying, and low fruit and vegetable intake, and had the highest prediabetes prevalence (27.3%).

Cluster-specific HbA1c distributions and differences in prediabetes prevalence are shown in Figure 2C and Figure 2D, respectively. Prediabetes prevalence differed significantly across dietary clusters (χ^2^ = 11.62, *p* = 0.003), indicating a graded association between overall dietary pattern quality and glycemic status.

### 3.3. Fast-Food Consumption, Dose–Response Patterns, and Prediabetes Risk

In binary logistic regression analysis, fast-food consumption frequency and BMI emerged as significant independent predictors of prediabetes (Table 2). Each unit increase in fast-food consumption score was associated with a 78% increase in the odds of prediabetes (OR = 1.78, 95% CI: 1.38–2.30, *p* < 0.001), independent of BMI, sex, and age. BMI was also positively associated with prediabetes risk (OR = 1.10 per kg/m^2^, 95% CI: 1.05–1.16, *p* < 0.001), whereas frying, sex, and age group were not significant predictors in the adjusted model.

Consistent with these findings, a clear dose–response relationship was observed between fast-food consumption frequency and glycemic markers (Table 3). Mean HbA1c increased progressively from 5.09% among students who reported never consuming fast food to 5.58% among those consuming fast food ≥ 3 times per week. Prediabetes prevalence rose in parallel, from 0.0% in non-consumers to 36.7% among frequent consumers, with a significant linear trend (Cochran–Armitage trend test: Z = 4.579, *p* < 0.001). Triglyceride concentrations showed a borderline positive trend across fast-food consumption categories (*p*-trend = 0.059).

### 3.4. BMI-Adjusted Associations and Mediation Analyses for HbA1c

Multivariable linear regression using HbA1c as a continuous outcome confirmed the independent associations observed in logistic models (Table 4). The overall model was statistically significant (F = 25.25, *p* < 0.001) and explained 18.1% of the variance in HbA1c (adjusted R^2^ = 0.174). Fast-food consumption frequency (β = 0.147, *p* < 0.001), fruit and vegetable intake (β = −0.109, *p* < 0.001), and BMI (β = 0.035, *p* < 0.001) were the strongest predictors, with comparable standardized effect sizes. Frying, sex, and age group did not show independent associations with HbA1c in the adjusted model.

**Figure 3:**
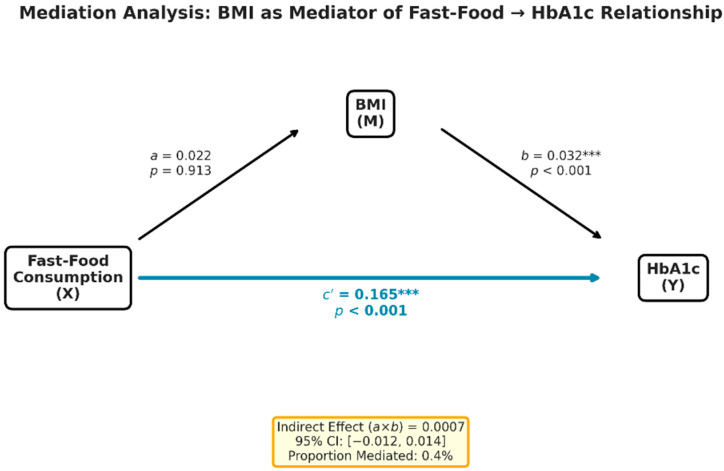
Path diagram of the mediation analysis, showing the non-significant indirect effect of fast-food consumption on HbA1c through BMI. ***—statistically significant, *p* < 0.001.

To evaluate whether BMI mediated the association between fast-food consumption and glycemic status, mediation analysis was performed. The indirect effect of fast-food consumption on HbA1c through BMI was not statistically significant (a × b = 0.0007; 95% CI: −0.012 to 0.014), accounting for only 0.4% of the total effect. The direct effect of fast-food consumption on HbA1c remained substantial after adjustment for BMI (c′ = 0.165), suggesting that the association is not primarily explained by differences in BMI (Figure 3).

**Figure 4:**
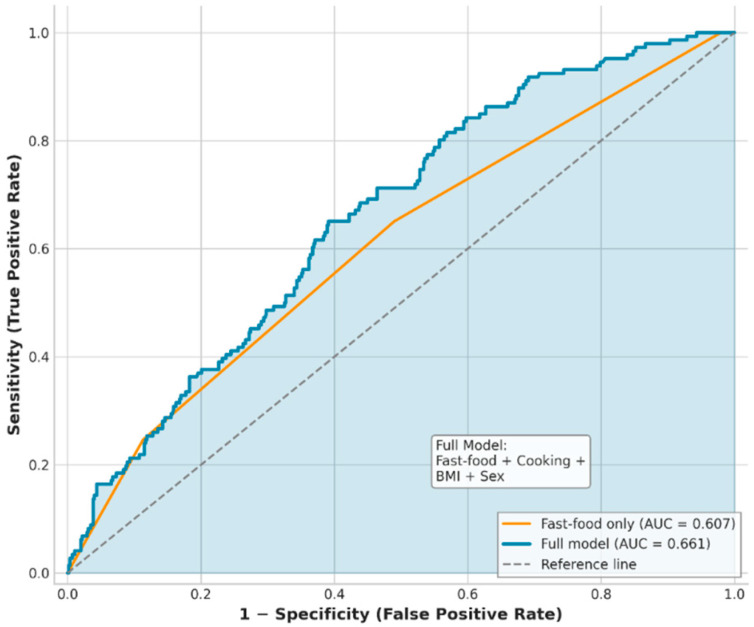
ROC curves for Prediabetes Prediction. ROC Curve Comparison of Fast-Food Consumption Alone Versus Multivariable Prediction Model for Prediabetes.

Receiver operating characteristic (ROC) curve analysis further contextualized these findings (Figure 4). A model including fast-food consumption alone demonstrated modest discriminatory ability for prediabetes (AUC = 0.607). Inclusion of BMI, cooking method, and sex resulted in only moderate improvement in predictive performance (AUC = 0.661), suggesting a limited incremental contribution of anthropometric variables beyond dietary exposure in this young population.

### 3.5. Lipid Profiles, Food Preferences, and TG/HDL Ratio

**Figure 5:**
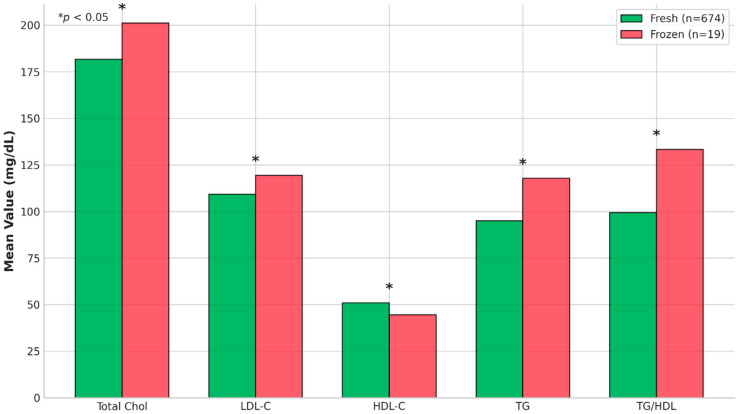
Lipid profile by food preference (fresh vs. frozen). Comparison of lipid profiles between students preferring fresh versus frozen foods.

In descriptive analyses, students reporting a preference for frozen foods (n = 19) exhibited higher total cholesterol, LDL-C, triglycerides, and TG/HDL ratio, and lower HDL-C compared with those preferring fresh foods (Figure 5). However, when food preference was included in multivariable models adjusting for dietary patterns, cooking method, BMI, sex, and age, frozen food preference was not independently associated with glycaemic or lipid outcomes. These findings should be interpreted cautiously due to the highly unbalanced group sizes.

In multivariable linear regression analyses of lipid parameters (Table 5), BMI was the only predictor consistently associated with all lipid outcomes. Fast-food consumption frequency was independently associated with higher triglyceride levels (β = 3.57 mg/dL, *p* = 0.034), while male sex was strongly associated with lower HDL-C (β = −10.04 mg/dL, *p* < 0.001). The mean TG/HDL ratio was 2.01, with 42.7% of participants exceeding the 2.0 threshold and 13.1% exceeding 3.0. In adjusted models, TG/HDL ratio was independently associated with BMI and male sex, but not with dietary variables.

### 3.6. Metabolic Outcomes Across Dietary Clusters: Stratified and Interaction Analyses

Stratified analyses were performed to evaluate whether the association between dietary clusters and glycemic status differed by sex or body mass index (BMI). Across all strata, the relationship between dietary patterns and HbA1c remained consistent. In both females and males, mean HbA1c values were lowest among participants in the Health-Conscious dietary cluster and highest in the Fast-Food Oriented cluster (ANOVA, *p* < 0.001) (Table 6 Panel A). A similar gradient was observed for the prevalence of prediabetes, with progressively higher rates across Health-Conscious, Mixed, and Fast-Food Oriented clusters in both sexes.

When stratified by BMI category, the association between dietary clusters and HbA1c remained statistically significant in both normal-weight (BMI < 25 kg/m^2^) and overweight/obese participants (BMI ≥ 25 kg/m^2^) (Table 6 Panel B). In both BMI strata, individuals adhering to the Health-Conscious cluster exhibited lower HbA1c values and a lower prevalence of prediabetes compared with those in the Mixed and Fast-Food Oriented clusters (*p* < 0.001 for normal-weight; *p* = 0.002 for overweight/obese participants). Markers of lipid metabolism, including the TG/HDL ratio, followed similar trends across dietary clusters, particularly among participants with overweight or obesity.

No meaningful effect modification by sex or BMI category was observed, indicating that the association between dietary patterns and glycemic markers was robust across these subgroups.

Interaction analysis revealed a statistically significant interaction between frying and high fast-food consumption for HbA1c (interaction β = −0.196, *p* = 0.023). Among students not using frying, high fast-food consumption was associated with a marked increase in HbA1c and prediabetes prevalence. Among frying users, HbA1c levels were already elevated in low fast-food consumers and did not increase further with higher fast-food intake, suggesting a ceiling effect (Table 7). No significant interaction was observed for TG/HDL ratio.

### 3.7. Sensitivity Analyses

Sensitivity analyses excluding participants with HbA1c ≥ 6.5% (n = 10) confirmed the robustness of the primary findings (Table 8). Associations between fast-food consumption, fruit and vegetable intake, BMI, and both continuous HbA1c and prediabetes risk remained statistically significant, with only minimal attenuation of effect sizes. These results indicate that the observed associations reflect early metabolic alterations rather than being driven by a small number of individuals with overt diabetes.

## 4. Discussion

Diet quality in early adulthood is an important determinant of long-term metabolic health. In young populations, dietary exposures that promote early dysglycemia may influence cardiometabolic risk as well as cognitive and emotional outcomes. Higher HbA1c levels have been associated with less favorable brain health and cognitive trajectories [27,36], while greater consumption of ultra-processed and fast-food dietary patterns has been linked to depressive and anxiety symptoms [22].

Within this context, our cross-sectional analysis of Romanian university students demonstrates a clear clustering of dietary behaviors that maps onto early glycemic vulnerability. Students adhering to a Fast-Food Oriented pattern exhibited nearly double the prevalence of prediabetes compared with those in the Health-Conscious pattern (27.3% vs. 15.4%). These findings align with the literature linking Westernized and ultra-processed dietary exposure to increased diabetes risk and extend this evidence by showing that, even within a narrow age range and a university setting, habitual constellations of food choices and preparation practices translate into measurable differences in HbA1c distribution [14]. Multiple analytic approaches in this study converged on the same pattern, with students reporting more frequent fast-food consumption consistently exhibiting higher HbA1c values and a greater prevalence of prediabetes, strengthening the interpretation that habitual dietary patterns are metabolically relevant in early adulthood, well before overt clinical disease becomes common [15].

The emergence of these patterns in a young, predominantly healthy population underscores early adulthood as a sensitive life-course period during which dietary behaviors may influence long-term metabolic trajectories. Supporting this interpretation, meta-analytic evidence indicates a dose–response association between ultra-processed food consumption and incident type 2 diabetes, lending plausibility to the graded associations observed between fast-food exposure and HbA1c in our cohort [13,37].

Although the cross-sectional design precludes causal inference, the concordance between dietary clustering, regression findings, and the observed dose–response pattern supports the interpretation that frequent fast-food exposure is a meaningful correlate of early dysglycemia in this age group.

The prediabetes prevalence observed in our cohort (21.1%) is important for a university-based young adult sample. Its magnitude exceeds estimates reported in some European university populations while resembling figures described in populations undergoing rapid nutritional transition. In a recent meta-analysis, the pooled prevalence of prediabetes across diverse settings was approximately 15% (95% CI: 13–18%), with particularly high estimates in urbanizing and transitional contexts [38].

These findings situate our results within the broader context of dietary and lifestyle transitions in Eastern and Central Europe, where increasing availability of convenience and ultra-processed foods may be reshaping metabolic risk profiles at younger ages. Consistent with this interpretation, nationally representative data in young adults aged 20–39 years indicate that prediabetes is increasingly detected and that behavioral and lifestyle factors contribute to early glycemic dysregulation alongside anthropometric risk [39].

While prevalence estimates vary by country, screening strategy, and biomarker definition, our HbA1c-based findings support the concept of “early glycemic risk” in young adults, which may be underestimated when screening strategies are limited to individuals with overweight or obesity [39]. Longitudinal evidence further indicates that higher consumption of ultra-processed foods is associated with impaired glucose homeostasis and increased risk of prediabetes and insulin resistance in young adult populations [40].

Against this backdrop, our biomarker-linked findings show that the university years may represent a practical window for prevention. This life stage is characterized by increasing autonomy over food choices within environments rich in convenience and ultra-processed foods, and accumulating evidence suggests that dietary patterns established during young adulthood can exert early metabolic effects. Population-based studies consistently show that higher fast-food and ultra-processed food intake is associated with impaired glucose regulation and prediabetes risk, even among young adults [41,42]. Accordingly, relatively modest improvements in dietary habits during this period may translate into meaningful differences in HbA1c at the population level.

Across analytic models, fast-food frequency remained a strong predictor of glycemic outcomes, whether prediabetes was modeled categorically or HbA1c analyzed continuously. The persistence of this association after adjustment for BMI and other covariates suggests that dietary behaviors themselves may contribute to early glycemic variation rather than simply reflecting adiposity differences. The regression analyses also revealed graded associations below the diagnostic prediabetes threshold, indicating that dietary behaviors may influence glycemic regulation well before clinically actionable cut points are reached. Similar continuous relationships between diet quality, lifestyle factors, and glycemic biomarkers have been reported in nationally representative samples of young adults [39]. Although the present study focuses on Romanian university students, the observed associations are broadly consistent with findings reported in other populations. Dietary patterns characterized by higher consumption of processed foods and lower intake of fruits and vegetables have been associated with less favorable metabolic profiles, including higher glycemic markers and cardiometabolic risk factors [43]. Similarly, cross-sectional research in Central and Eastern European populations has reported that dietary patterns rich in energy-dense and processed foods are associated with higher prevalence of diabetes and metabolic disturbances [44]. While cultural dietary patterns differ, the consistency of these associations across studies supports the relevance of our findings for young adult populations beyond Romania.

From a clinical and public health perspective, such subclinical glycemic shifts are important because higher HbA1c levels, even within non-diabetic ranges, have been associated with increased cardiovascular risk, and prediabetes itself contributes elevated long-term risks of macrovascular outcomes and mortality [41]. In parallel, greater exposure to ultra-processed foods has been linked to deterioration in glucose homeostasis and higher odds of prediabetes in longitudinal studies [40]. Experimental feeding studies further support biological plausibility, showing that manipulating the degree of food processing can rapidly affect cardiometabolic physiology [45]. These findings are consistent with evidence that metabolic alterations related to ultra-processed food exposure may be detectable in biochemical profiles even outside overt disease states [46].

In contrast to fast-food exposure, fruit and vegetable intake showed an independent inverse association with HbA1c, suggesting that overall dietary quality may be particularly relevant in this setting. The negligible indirect effect observed in mediation analysis indicates that the association between fast-food consumption and HbA1c is not primarily explained by differences in BMI. Instead, dietary exposures may influence glucose regulation through mechanisms such as glycemic load, food-processing characteristics, or metabolic responses to dietary additives. This interpretation aligns with epidemiological evidence showing that fast food and ultra-processed foods are associated with dysglycemia and diabetes risk even after adjustment for BMI [47].

From a dietary-processing perspective, such patterns may influence glycemic regulation through characteristics including high glycemic load, low fiber content, reduced satiety, and food additives that may affect gut and metabolic pathways [47,48]. Emerging evidence implicates the microbiota–gut–brain axis as a potential pathway linking dietary patterns with metabolic and neurobehavioral outcomes. Diets rich in ultra-processed foods and low in dietary fiber may alter gut microbial composition, disrupt microbial metabolite production, and promote systemic inflammation, processes that may impair insulin signaling and glucose regulation [49,50]. Experimental and translational studies further suggest that microbial activity influences host insulin sensitivity and metabolic homeostasis [51], while gut-derived inflammatory and endocrine signals may also affect brain function through immune, neural, and hormonal pathways [52,53].

The observation that normal-weight students within the Fast-Food Oriented cluster still exhibited substantial prediabetes prevalence mirrors the metabolically unhealthy normal-weight phenotype, which has been associated with elevated diabetes risk despite normal BMI [54]. These findings support prevention strategies that do not rely exclusively on anthropometric screening.

Cooking practices contributed an additional clinically relevant layer. High-heat cooking methods such as frying and grilling were associated with higher HbA1c, a finding consistent with evidence that high-temperature cooking increases dietary advanced glycation end products and related compounds that promote oxidative stress and inflammation [55]. Controlled dietary interventions further demonstrate that modifying cooking methods can reduce AGE exposure and related metabolic biomarkers in humans [56].

Our interaction analysis suggested a ceiling-type pattern: among frequent frying users, HbA1c was already elevated even at lower levels of fast-food intake, whereas among non-frying users, high fast-food consumption was associated with a more pronounced HbA1c increase. This pattern suggests that frequent exposure to high-heat cooking practices may elevate glycemic markers to a level where additional fast-food intake produces only limited further change, potentially reflecting cumulative exposure to multiple glycemic stressors in dietary environments where fried foods and convenience meals frequently co-occur. Experimental and observational evidence confirms that frying substantially increases AGE content and lipid oxidation products, which have been linked to insulin resistance and impaired glucose metabolism [21,57]. Residual confounding remains a possible explanation, as frying frequency may proxy a broader home food environment characterized by lower overall diet quality and correlated behaviors [14]. Nevertheless, the interaction is clinically suggestive, highlighting cooking-method modification as an accessible intervention lever in student populations.

Regarding lipid-related outcomes, the TG/HDL ratio indicated a substantial burden of subclinical cardiometabolic vulnerability in this cohort; however, after adjustment, BMI and sex were the dominant predictors, with weaker independent dietary associations. The weaker dietary associations observed for lipid parameters compared with glycemic markers may indicate that early dietary perturbations in young adults are first reflected in glucose regulation, with lipid abnormalities potentially emerging later in the trajectory of metabolic dysfunction. This interpretation is consistent with evidence that TG/HDL is a useful insulin-resistance-related marker but is strongly shaped by adiposity and sex-related metabolic differences [58,59,60,61]. The independent association between fast-food frequency and triglycerides, but not LDL-cholesterol or total cholesterol, aligns with metabolic signatures linked to refined carbohydrate and ultra-processed dietary patterns [35,49,62,63,64,65,66].

Finally, the descriptive signal related to frozen food preference diminished in multivariable models and should be interpreted cautiously given the small subgroup size. This likely reflects broader dietary patterning rather than a direct effect of food storage and highlights the need for future studies to distinguish frozen minimally processed foods from frozen ultra-processed convenience products. Although psychological and cognitive outcomes were not assessed in this study, converging evidence linking higher HbA1c and prediabetes to adverse cognitive trajectories [27], and ultra-processed food exposure to mental health symptoms [23], reinforces the potential value of early dietary prevention strategies in young adults. From a campus-health perspective, relatively simple targets—reducing fast-food frequency, increasing fruit and vegetable intake, and lowering reliance on high-heat frying—may shift HbA1c at the population level with broader health benefits [67,68,69]. These preventive strategies may also be biologically relevant given the central role of oxidative stress and inflammation in metabolic and gastrointestinal disease processes, which are increasingly recognized as downstream pathways of diet-related metabolic dysregulation [22]. Taken together, these findings highlight several modifiable behaviors, particularly frequent fast-food consumption, low fruit and vegetable intake, and reliance on high-heat cooking methods, which may represent practical targets for early metabolic risk reduction in university populations.

Several limitations should be considered when interpreting these findings. The cross-sectional design precludes causal inference, and longitudinal studies are needed to confirm temporal relationships between dietary exposures and metabolic outcomes. Dietary data were self-reported and may be subject to recall or social desirability bias. The convenience sampling strategy limits generalizability beyond Romanian medical university students. Physical activity and sleep patterns were not assessed and may represent important confounding variables. Additionally, the highly unbalanced distribution of frozen food preference limited statistical power for evaluating this variable. Another limitation is that overweight and obese participants were grouped into a single BMI category (BMI ≥ 25 kg/m^2^), which may have limited the ability to explore potential differences in metabolic associations between overweight and obese individuals. Finally, the use of categorical age groups rather than continuous age values reduced adjustment precision [70,71,72].

Despite these limitations, this study has several strengths. The relatively large sample size, inclusion of objective biochemical measurements, and application of advanced statistical methods (including cluster analysis, mediation modeling, dose–response analysis, stratified regression, and sensitivity analyses) provide a comprehensive evaluation of dietary influences on early metabolic risk. To our knowledge, this is the first study to examine the combined effects of dietary patterns and cooking methods on prediabetes risk among Romanian university students.

## 5. Conclusions

This study identified significant associations between unfavorable dietary patterns, especially frequent fast-food consumption and reliance on high-temperature cooking methods, and elevated glycemic risk markers among Romanian university students. A clear dose–response relationship between fast-food consumption frequency and HbA1c was observed, suggesting that differences in dietary habits may already be reflected in early glycemic variation in young adults.

These associations were consistent across sex and BMI categories and remained robust after excluding participants with overt diabetes. Importantly, normal-weight students adhering to fast-food-oriented dietary patterns also exhibited substantial prediabetes prevalence, indicating that early metabolic vulnerability may occur even among individuals with normal BMI. Higher fruit and vegetable intake showed an independent inverse association with HbA1c, highlighting the potential relevance of overall dietary quality in early adulthood.

Collectively, these findings highlight dietary behaviors that may be relevant for early metabolic risk profiles in young adults. From a public health perspective, strategies promoting healthier food choices and cooking practices during the transition to independent living may represent promising targets for preventive interventions. Future longitudinal studies are needed to confirm these associations and clarify the temporal and mechanistic pathways linking dietary exposures to early glycemic alterations.

## Figures and Tables

**Table 1:** Baseline characteristics of study participants (n = 693).

Variable	Descriptive Statistics
Sample Volume	693
Age, 21–24 years (%)	58.0
Female (%)	58.6
BMI (kg/m^2^), mean ± SD	23.96 ± 3.86
Total Cholesterol (mg/dL), mean ± SD	182.1 ± 31.3
LDL Cholesterol (mg/dL), mean ± SD	109.6 ± 21.4
HDL Cholesterol (mg/dL), mean ± SD	50.8 ± 12.4
Triglycerides (mg/dL), mean ± SD	95.6 ± 33.7
HbA1c (%), mean ± SD	5.34 ± 0.48
Prediabetes, n (%)	146 (21.1)
Diabetes, n (%)	10 (1.4)

**Table 2:** Binary logistic regression predicting prediabetes risk.

Variable	Odds Ratio	95% CI	*p*-Value
Fast-food score	1.78	1.38–2.30	<0.001
Frying (vs. other)	1.26	0.81–1.95	0.311
BMI (per unit)	1.10	1.05–1.16	<0.001
Male (vs. female)	0.86	0.58–1.27	0.447
Age 21–24 (vs. 18–20)	0.91	0.62–1.33	0.612

**Table 3:** Dose–response relationship between fast-food consumption and metabolic markers.

Fast-Food Frequency	n	HbA1c (%) Mean ± SD	Prediabetes n (%)	Triglycerides (mg/dL) Mean ± SD
Never	11	5.09 ± 0.35	0 (0.0%)	104.9 ± 28.6
Rarely	319	5.23 ± 0.48	51 (16.0%)	93.3 ± 32.4
1–2 times/week	265	5.38 ± 0.44	59 (22.3%)	95.3 ± 33.6
≥3 times/week	98	5.58 ± 0.49	36 (36.7%)	102.8 ± 37.5
*p*-trend		<0.001	<0.001	0.059

**Table 4:** Multivariable linear regression predicting HbA1c (%) (n = 693).

Variable	β	95% CI	*p*-Value	Std. β
Fast-food frequency (0–3)	0.147	0.102, 0.192	<0.001	0.225
Frying (vs. other methods)	0.054	−0.027, 0.135	0.190	0.046
Fruit/vegetable intake (0–3)	−0.109	−0.144, −0.075	<0.001	−0.217
BMI (kg/m^2^)	0.035	0.026, 0.044	<0.001	0.283
Male sex	−0.048	−0.118, 0.022	0.178	−0.049
Age 21–24 (vs. 18–20)	−0.015	−0.081, 0.051	0.652	−0.016

Model: F(6686) = 25.25, *p* < 0.001; R^2^ = 0.181, adjusted R^2^ = 0.174.

**Table 5:** Multivariable linear regression for lipid parameters (n = 693).

Predictor	TC β (*p*)	LDL-C β (*p*)	HDL-C β (*p*)	TG β (*p*)
Fast-food frequency	−0.92 (0.548)	−0.63 (0.553)	0.39 (0.499)	3.57 (0.034)
Frying	−2.69 (0.327)	−1.53 (0.423)	−0.82 (0.423)	−0.80 (0.792)
Fruit/vegetable intake	−0.88 (0.455)	−0.47 (0.561)	0.30 (0.496)	1.44 (0.265)
BMI	3.10 (<0.001)	1.90 (<0.001)	−0.58 (<0.001)	2.82 (<0.001)
Male sex	−0.22 (0.928)	−0.96 (0.562)	−10.04 (<0.001)	−1.30 (0.619)
Age 21–24	−2.02 (0.367)	−1.69 (0.279)	−0.81 (0.334)	−0.27 (0.914)
R^2^	0.147	0.116	0.239	0.110

**Table 6:** Metabolic markers by dietary cluster, stratified by sex and BMI category. Panel A. Stratified by Sex.

**Panel A. Stratified by Sex**				
**Variable**	**Health-Conscious**	**Mixed**	**Fast-Food Oriented**	***p*-Value**
Females (n = 406)				
n	174	81	151	<0.001
HbA1c (%)	5.18 ± 0.50	5.37 ± 0.45	5.43 ± 0.48	
Prediabetes (%)	13.8	22.2	25.8	
Total cholesterol (mg/dL)	179.5	177.5	179.5	
HDL cholesterol (mg/dL)	54.8	55.2	56.5	
Triglycerides (mg/dL)	91.5	90.0	96.7	
Males (n = 287)				
n	106	64	117	<0.001
HbA1c (%)	5.21 ± 0.41	5.50 ± 0.47	5.46 ± 0.46	
Prediabetes (%)	11.3	29.7	29.1	
Total cholesterol (mg/dL)	190.3	183.7	184.2	
HDL cholesterol (mg/dL)	44.8	43.2	44.1	
Triglycerides (mg/dL)	95.2	102.4	100.6	
**Panel B. Stratified by BMI Category**				
**Variable**	**Health-Conscious**	**Mixed**	**Fast-Food Oriented**	***p*-Value**
Normal weight (BMI < 25 kg/m^2^, n = 444)				
n	184	97	163	<0.001
HbA1c (%)	5.11 ± 0.44	5.36 ± 0.44	5.36 ± 0.46	
Prediabetes (%)	9.2	18.6	22.7	
TG/HDL ratio	1.74	1.73	1.85	
Overweight/Obese (BMI ≥ 25 kg/m^2^, n = 249)				
n	96	48	105	0.002
HbA1c (%)	5.35 ± 0.47	5.56 ± 0.47	5.57 ± 0.46	
Prediabetes (%)	19.8	39.6	34.3	
TG/HDL ratio	2.31	2.75	2.36	

Values are presented as mean ± standard deviation or percentage, as appropriate. *p*-values refer to one-way ANOVA comparing dietary clusters within each stratification group.

**Table 7:** Mean HbA1c and prediabetes prevalence by frying and fast-food consumption.

Frying Status	Fast-Food Consumption	n	HbA1c (%) Mean ± SD	Prediabetes (%)
No frying	Low	280	5.19 ± 0.46	12.9
	High	268	5.44 ± 0.47	27.2
Frying	Low	50	5.44 ± 0.49	30.0
	High	95	5.42 ± 0.45	23.2

The interaction term for TG/HDL ratio was not significant (β = −0.105, *p* = 0.515), indicating that the metabolic interplay between cooking method and fast-food frequency may be specific to glycemic rather than lipid markers.

**Table 8:** Comparison of key regression estimates: full sample vs. sensitivity analysis.

Parameter	Full Sample (n = 693)	Sensitivity (n = 683)
Linear regression (HbA1c continuous)		
Fast-food β (*p*)	0.147 (<0.001)	0.136 (<0.001)
Fruit/vegetable β (*p*)	−0.109 (<0.001)	−0.105 (<0.001)
BMI β (*p*)	0.035 (<0.001)	0.032 (<0.001)
R^2^	0.181	0.173
Logistic regression (prediabetes)		
Fast-food OR (95% CI)	1.78 (1.38–2.30)	1.66 (1.28–2.17)
Fruit/vegetable OR (95% CI)	—	0.63 (0.51–0.76)
BMI OR (95% CI)	1.10 (1.05–1.16)	1.11 (1.06–1.17)

The minimal attenuation of effect sizes (e.g., fast-food β: 0.147 → 0.136) confirms that observed associations are not driven by a small number of individuals with overt diabetes but reflect genuine early metabolic alterations in an ostensibly healthy young population.

## Data Availability

The data presented in this study are available on request from the corresponding author. The data are not publicly available due to privacy and ethical restrictions.

## Supplementary Materials

The following supporting information can be downloaded at: https://www.mdpi.com/article/10.3390/nu18060977/s1, File S1: Questionnaire.

## Abbreviations

The following abbreviations are used in this manuscript:

AGE	Advanced glycation end product
BMI	Body Mass Index
CI	Confidence interval
HbA1c	Glycated hemoglobin
HDL-C	High-density lipoprotein cholesterol
IDF	International Diabetes Federation
LDL-C	Low-density lipoprotein cholesterol
OR	Odds ratio
ROC	Receiver operating characteristic
SD	Standard deviation
SEM	Standard error of the mean
T2DM	Type 2 diabetes mellitus
TG	Triglycerides
TG/HDL	Triglyceride-to-HDL cholesterol ratio
UPF	Ultra-processed foods
