# Supplementary material for: Dietary Patterns, Cooking Methods, and Their Association with Prediabetes Risk Markers in Romanian University Students: A Cross-Sectional Analysis
Source: Nutrients. 2026 Mar 19;18(6):977. doi: 10.3390/nu18060977 (PMC13028893; doi:10.3390/nu18060977)
Supplement: Supplementary file 1 [file nutrients-18-00977-s001.zip › nutrients-4194118-supplementary.pdf]

Title: Study on Lifestyle, Nutrition, and Health Indicators

Section 1: Participation Agreement (Informed Consent)

This section must be the first page of the form. Use the "Go to next section" option only after the respondent confirms their consent.
Information for participants: You are invited to participate in a research study regarding the correlation between eating habits and biochemical indicators.
Privacy: Participation is anonymous. No names, email addresses (except for Google sign-in process if enabled, but these will not be linked to the responses), contact details or professional identifiers are collected.
Data collection: Collected data are anonymous and do not allow any direct or indirect identification of participants. No names, initials, contact details or other information that could lead to the identification of the person will be collected. All data will be analyzed and reported only in aggregate form. Provided information will be stored securely and confidentially, in compliance with applicable personal data protection legislation (General Data Protection Regulation – GDPR, EU 2016/679). This study is observational in nature and does not involve any additional medical intervention. Completing the questionnaire does not entail medical risks and does not replace specialist medical recommendations or consultations.
Data processing: Responses will be analyzed exclusively in aggregate (group-level). However, please note that theoretically, specific combinations of demographic variables could allow indirect identification.
Volunteering: Participation is voluntary and you may withdraw at any time by closing the form. By completing this questionnaire, you agree that your responses will be used solely for scientific purposes, within academic analyses, doctoral thesis, and scientific articles.
Participants' agreement: By continuing and completing the questionnaire, you confirm that:
- you have read and understood the above information;
- you agree to participate voluntarily in this study;
- you allow the anonymous use of data strictly for scientific purposes.

☐ I agree to participate in this study and to complete the questionnaire.
Question 1 (Mandatory): I confirm that I have read the above information and agree to participate in this study.
[ ] Yes, I agree.
Question 2 (Mandatory): Please enter today's date to validate your consent.
Answer type: Date (DD/MM/YYYY)

Section 2: Demographic and Anthropometric Profile
Gender:
[ ] Female
[ ] Male

Age (range):
[ ] 18 - 20 years
[ ] 21 - 25 years
[ ] [Other ranges...]
Height (cm): (e.g., 175)
Current weight (kg):

Section 3: Eating Habits and Lifestyle
How many times do you eat per day?
What is your main meal of the day?
[ ] Breakfast
[ ] Lunch
[ ] Dinner
Do you eat breakfast every morning? (Yes/No)
Do you believe breakfast is the most important meal of the day? (Yes/No)
Are you vegetarian? (Yes/No)
What do you usually eat for lunch? (Multiple options: Meat, Vegetables/Salad, Fish, etc.)
Where do you usually have lunch? (At home, Canteen, Restaurant, etc.)
What kind of dinner do you usually have? (Hot/cooked, Cold/snack, etc.)
How much water do you drink per day? (Under 1L, 1L-2L, over 2L)
How many cups of tea or coffee do you drink per day?
How many times per week do you eat fruits and vegetables?
How often do you eat fast food?
What is the most common cooking method in your household? (Boiling, Frying, Baking, etc.)
Do you pay attention to the ingredients in the foods you buy?

Section 4: Clinical Indicators (If available)
Note: This section is for entering data from medical tests.
Total cholesterol (mg/dL):
LDL (mg/dL):
HDL (mg/dL):
Triglycerides (mg/dL):
HbA1c (%):
Weight recorded during previous monitoring (year 1), if applicable (kg):
Instructions for setting up in Google Forms:
Response validation: For numeric fields (height, weight, tests), enable “Response validation” -> “Number” -> “Is number” to ensure data accuracy.

Sectioning: Use the “Add section” button (two horizontal bars) to separate Consent from the rest of the questions.
Collection settings: In the “Settings” tab, make sure the option “Collect email addresses” is set to “Do not collect” to respect the anonymity promised in the consent text.
